# RD Lawrence Lecture 2008 Targeting GLP-1 release as a potential strategy for the therapy of Type 2 diabetes

**DOI:** 10.1111/j.1464-5491.2008.02514.x

**Published:** 2008-08

**Authors:** F M Gribble

**Affiliations:** Cambridge Institute for Medical Research and Department of Clinical Biochemistry, Wellcome Trust/MRC Building, Addenbrooke's HospitalCambridge, UK

**Keywords:** GLP-1, GIP, Incretin

## Abstract

Glucagon-like peptide-1 (GLP-1) and glucose-dependent insulinotropic polypeptide (GIP) are gastrointestinal hormones that play an important role in stimulating postprandial insulin release from pancreatic β-cells. Agents that either mimic GLP-1 or prevent its degradation are now available for the treatment of Type 2 diabetes, and strategies to enhance endogenous GLP-1 release are under assessment. As intestinal peptides have a range of actions, including appetite regulation and coordination of fat metabolism, harnessing the enteric endocrine system is a promising new field for drug development.

Diabet. Med. 25, 889–894 (2008)

Glucagon-like peptide-1 (GLP-1) and glucose-dependent insulinotropic polypeptide (GIP), are intestinal hormones secreted in response to food ingestion, which stimulate the release of insulin from pancreatic β-cells. Together they account for the finding that oral glucose is a more potent stimulus for insulin secretion than intravenous glucose—the so-called ‘incretin’ effect [1]. They are biologically and therapeutically interesting peptides, not only because of their potential anti-hyperglycaemic effects in Type 2 diabetes, but also because GLP-1 reduces appetite and GIP modulates how the body handles dietary fats.

The original finding that GLP-1 infusion increased insulin release and reduced fasting plasma glucose and glucagon in Type 2 diabetic subjects [2] resulted in the development of the first GLP-1 receptor agonists, and the recent licensing of exenatide for the treatment of Type 2 diabetes. Interestingly, longer-term studies have shown that chronic therapy with GLP-1 mimetics is also associated with beneficial effects on body weight [3]. As the active form of GLP-1 is normally degraded rapidly in the circulation by the enzyme dipeptidyl peptidase 4 (DPP-4), drug design in the field of GLP-1-based therapies is predominantly based around producing GLP-1 mimetics that are DPP-4 resistant, or pharmacologically inhibiting the enzymatic activity of DPP-4 (e.g. Sitagliptin, Vildagliptin) [4].

Both approaches have their drawbacks. The GLP-1 mimetics currently under development are peptide derivatives that are not orally available, and although a small molecule agonist of the GLP-1 receptor has been described [5], it is not clear whether similar small molecules could be developed that would be suitable for human therapeutics. The potential disadvantage of inhibiting DPP-4, by contrast, is that the enzyme is responsible for the cleavage of a wide range of plasma peptides, as well as having a role in the immune system, and the full biological consequences of its chronic inhibition remain uncertain. A third therapeutic strategy, currently under exploration, is to stimulate the endogenous release of GLP-1 from the intestinal L-cells. As proof of principle, it has recently been reported that a small molecule (AR231453), which targets GPR119 on L-cells, stimulates GLP-1 release and improves glucose tolerance in rodents, both on its own and additively with a DPP-4 inhibitor [6]. It has not, however, yet been demonstrated definitively that enhancing GLP-1 secretion in a subject with Type 2 diabetes would provide a sufficient stimulus to improve glycaemia.

## GLP-1 effects on pancreatic islet hormones

**FIGURE 1:**
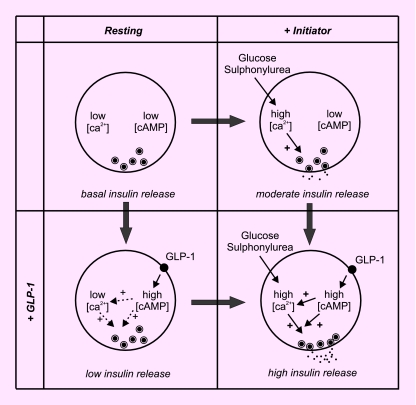
Glucose-dependent stimulation of insulin release by glucagon-like peptide-1 (GLP-1). At low glucose concentrations (left), intracellular [Ca^2+^] is low, and although stimulation by GLP-1 causes an increase in cyclic adenosine monophosphate (cAMP), this causes only small increases in [Ca^2+^] and minimal enhancement of insulin release (dashed arrows). The amount of insulin released is not enough to induce hypoglycaemia. At high glucose concentrations or in the presence of sulphonylureas (right), [Ca^2+^] is elevated, and the elevation of cAMP by GLP-1 can then potently enhance the rate of insulin release, as well as further increasing [Ca^2+^].

The principal therapeutic action of GLP-1 arises from its ability to stimulate insulin release in a glucose-dependent manner. It also stimulates β-cell proliferation and reduces β-cell apoptosis [7], although it remains to be established whether these actions contribute to the effectiveness of GLP-1 mimetics *in vivo*. The glucose-dependence of GLP-1 can be understood by considering insulin secretagogues as either initiators or amplifiers of secretion (Fig. 1). Whereas initiators, such as glucose, trigger insulin release by depolarizing the plasma membrane and enabling an influx of calcium that signals to the secretory vesicles to release their contents, amplifiers only enhance secretion after it has first been initiated. In the cases of GLP-1 and GIP, this is through their binding to specific G-protein-coupled receptors on the β-cell membrane, which activate adenylate cyclase and increase cytoplasmic cyclic adenosine monophosphate (cAMP) levels. cAMP has a range of actions in the β-cell, but probably enhances insulin release via pathways dependent on both protein kinase A and Epac proteins [8]. The physiological interaction between initiators and amplifiers of insulin release is important therapeutically, because sulphonylureas are initiators of insulin release, and their action can therefore be enhanced by GLP-1. In patients taking sulphonylureas (or their equivalents) the glucose dependence of GLP-1 is lost [9], and the combination therapy can result in hypoglycaemic side effects.

In addition to the stimulation of insulin release, GLP-1 also inhibits glucagon release and stimulates somatostatin secretion [10]. Indeed, the inhibition of glucagon release by GLP-1 is believed to contribute to the clinical effectiveness of GLP-1 mimetics for the treatment of Type 2 diabetes, as the hyperglucagonaemia commonly seen in diabetes is partially responsible for the raised glucose levels. Exactly how GLP-1 inhibits glucagon release is unclear. GLP-1 receptors have been detected in at least a subset of pancreatic α-cells, and it has been confirmed that GLP-1 dose-dependently inhibits glucagon secretion from perfused pancreas preparations [10]. Yet the GLP-1 receptor in α-cells is believed to be identical to that in β-cells, and its activation should similarly increase cAMP concentrations. As elevated cAMP levels in α-cells, as in β-cells, are generally believed to increase, not decrease, hormone release [11], the inhibitory nature of GLP-1 on glucagon secretion currently remains a paradox.

Despite the demonstration that GLP-1 receptors are expressed in pancreatic β-cells and that GLP-1 stimulates insulin release from islets and insulinoma cell lines *in vitro*, it is by no means certain that the entire physiological effect of endogenously secreted GLP-1 is mediated by direct interaction of the hormone with the islet cells. GLP-1 has a circulating half-life of 1–2 min and is rapidly inactivated by DPP-4 in the splanchnic bed and liver. The levels of active GLP-1 that reach the pancreas are therefore significantly lower than those in the portal circulation. Furthermore, even after a meal, the circulating levels of GLP-1 are at the low end of the range needed to stimulate β-cells, and supraphysiological concentrations of exogenously infused GLP-1 are employed to stimulate insulin release in Type 2 diabetic subjects. Nevertheless, the relatively modest elevation of circulating active GLP-1 levels achieved by inhibiting DPP-4 has been clearly demonstrated to be therapeutically useful in diabetes. These findings raise the possibility that locally released GLP-1 may be more effective than peripherally administered GLP-1, and that the primary target of GLP-1 from the entero-endocrine cells may reside closer to the site of its secretion within the splanchnic circulation. In support of this idea, GLP-1 receptors have been detected immunohistologically in human portal vein sections, with corresponding GLP-1 receptor mRNA expression in the nodose ganglion (the inferior ganglion of the vagus nerve) [12], suggesting that GLP-1 receptors are expressed in afferent branches of the vagus, including those innervating the portal vein. However, the functional role of GLP-1 receptors within the alimentary tract and portal vein, and their relative contribution to the incretin effect compared with those located on the pancreatic β-cell itself, remain to be established.

## GLP-1 effects on appetite

Since the early trials of GLP-1 in human subjects, it has been apparent that the hormone not only increases insulin release but also reduces appetite. Although a portion of the effect was initially attributed to nausea associated with reduced gastric emptying, it is now apparent that GLP-1 also acts on central appetite circuits. GLP-1 receptors have been identified in regions of the hypothalamus such as the arcuate nucleus [13], which are known to be involved in appetite regulation, and it is possible that these may respond to changes in circulating GLP-1 levels. GLP-1 is also produced by subgroups of brain stem neurons that have been shown to be activated by gastric distension, and that project, in part, to the hypothalamus [13,14]. The role of central GLP-1 and its importance for appetite control and glucose homeostasis remain to be fully elucidated.

## GLP-1 secretion

GLP-1 is one of a family of hormones released from specialized entero-endocrine cells situated in the epithelial layer of the gastrointestinal tract. GLP-1-producing L-cells have been identified in the jejunum, ileum and colon, with the highest cell densities reported in the ileum and colon. The cells have an apical membrane surface facing into the gut lumen, which may be involved in sampling the passing nutrients, and secretory vesicles located towards the basolateral surface. Together in the same cells, and even in the same vesicles, it has been shown that GLP-1 may colocalize with Peptide YY in the colon, and with GIP in upper regions of the small intestine. Whether this is the only difference between the GLP-1-producing cells from different gut regions remains to be established. In all cells producing GLP-1, there is also concomitant production of GLP-2 and oxyntomodulin, as all three molecules are formed when the proglucagon precursor is cleaved by prohormone convertase 1/3. In pancreatic α-cells, by contrast, prohormone convertase 2 cleaves the same proglucagon molecule to produce glucagon and a major proglucagon fragment containing the GLP-1 and GLP-2 sequences.

The principal stimulus for GLP-1 release is food ingestion. Elevation of peripheral GLP-1 levels can be detected within 10–15 min of eating, and persists for up to several hours, depending on the nutritional composition of the meal. The time-dependence of GLP-1 secretion may reflect, at least in part, the corresponding changes in nutrient levels at different regions of the gut, as the food reaches, and stimulates, the different populations of L-cells. However, several studies have concluded that the early rise in GLP-1 following a meal includes GLP-1 secreted from distal L-cells, which are stimulated by neural or hormonal signals arising from proximally located nutrient sensors.

In most studies on Type 2 diabetic subjects, GLP-1 levels are reduced [15]. The commonest finding is that the later phase of GLP-1 elevation is impaired in diabetes, but reduced fasting levels have also been found in some studies. The mechanism underlying impaired GLP-1 secretion has not been established, but evidence suggests that it is a secondary consequence of the diabetic state, as it was not, for example, found in first-degree relatives of Type 2 diabetic subjects or in people with a previous history of gestational diabetes [16]. How diabetes affects the L-cells, and whether the cells exhibit adaptive changes in their density, distribution or function, will be interesting areas for future research.

Like other intestinal epithelial cells, L-cells have a relatively rapid turnover. They originate from pluripotent stem cells in the crypts, differentiate as they migrate out of the crypts and up the villi, and are shed from the villous tips after a lifetime of 5–7 days. This life cycle has interesting implications for strategies aimed at targeting the cells as a therapy for Type 2 diabetes. It seems likely that even if the L-cells are damaged acutely by the metabolic manifestations of diabetes, reversal of the metabolic abnormalities would be accompanied by restoration of normal GLP-1 secretion, as the previously damaged L-cells would be replaced by a new L-cell population. Unless the crypt stem cell population has been permanently damaged or there is a primary defect in the L-cells, it would seem likely that L-cells in metabolically controlled diabetes would be as responsive to therapeutic stimulation as the corresponding cells in healthy subjects.

## Mechanisms underlying GLP-1 secretion: *in vivo* and perfused gut studies

A number of studies addressing the pathways involved in GLP-1 secretion have involved feeding humans or animals with a range of nutrients, whilst measuring GLP-1 in the peripheral circulation or portal vein. In other experiments, isolated perfused segments of gut have been studied in anaesthetized animals, and the GLP-1 concentrations measured in response to nutrients instilled into the gut lumen, or to hormones and neurotransmitters perfused through the mesenteric circulation. Such studies have shown that GLP-1 secretion is triggered by glucose, fats and proteins, and that the plasma levels reflect both the calorific value and the nature of the ingested nutrients [16]. Responses to monosaccharides have been shown to be sodium-dependent, and not to require metabolism of the sugar [17]; fats were found to be more effective when they contained longer unsaturated acyl chains; and peptones as well as amino acid mixtures replicated the secretory responses to proteins. GLP-1 release was also triggered by hormones such as gastrin-releasing peptide (the mammalian equivalent of bombesin) and GIP (in mice, but not humans), as well as by acetylcholine acting through muscarinic receptors.

## Mechanisms underlying GLP-1 secretion: *in vitro* studies

GLP-1 secretion has been investigated *in vitro* using a variety of cellular models, including fetal rat intestinal cell cultures, canine intestinal cell cultures enriched for L-cells by elutriation, and cell lines such as GLUTag, STC-1 and NCI-H716. Unfortunately, it has been remarkably difficult to establish reliable primary cultures of adult rodent intestinal cells suitable for studies of enteroendocrine cell function. Of the two murine cell lines, GLUTag and STC-1, the former is perhaps the more reliable model of GLP-1 release, as it was established from a colonic tumour from a transgenic mouse expressing SV40 large T antigen driven by the proglucagon promoter [18]. STC-1 cells were originally developed as a model of secretin secretion and secrete a wide range of enteric hormones in addition to GLP-1. NCI-H716 is notable as the only human GLP-1-secreting cell line currently available. Only a limited number of studies have been reported using this cell type.

Studies on these L-cell models have yielded both consistent and conflicting results. It is a uniform finding, for example, that elevating cytoplasmic cAMP is a potent stimulus for GLP-1 release, whereas the effectiveness of changing the nutrient concentrations is more variable. The robust responsiveness to cAMP suggests that signals (hormonal, neural or nutritional) coupled through G-protein-coupled receptors linked to adenylate cyclase might similarly trigger GLP-1 release *in vivo*. Establishing whether L-cells also respond directly to nutrients such as glucose is, however, critical to our understanding of the overall behaviour of the entero-endocrine system.

**FIGURE 2:**
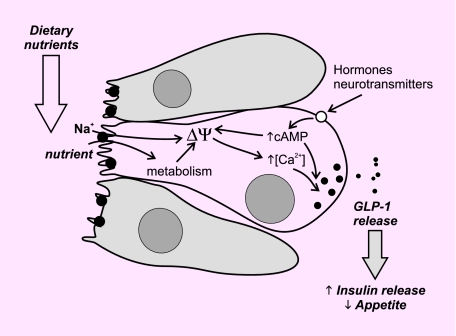
A working model of glucagon-like peptide-1 (GLP-1) release from the intestinal L-cell. Dietary nutrients stimulate GLP-1 release from intestinal L-cells, as a consequence of their Na^+^-coupled uptake and subsequent metabolism, which cause membrane depolarization (ΔΨ) and Ca^2+^ entry. Indirect signals from hormones and neurotransmitters can stimulate release by a variety of mechanisms, including elevation of cyclic adenosine monophosphate (cAMP) (which acts as both an initiator and an amplifier of GLP-1 release), and Ca^2+^ release from stores. GLP-1 released at the basolateral membrane stimulates insulin release from pancreatic β-cells and reduces appetite.

GLUTag cells are electrically active, and fire action potentials when stimulated with nutrients or raised cAMP concentrations [19,20]. The coupling between nutrient changes and hormone secretion is analogous to that found in other endocrine cells, involving membrane depolarization, calcium entry and calcium-dependent vesicle release (Fig. 2). As in pancreatic β-cells, agents that stimulate GLUTag cells can act as initiators and/or amplifiers of GLP-1 release, raising the possibility that L-cells *in vivo* may integrate signals from a variety of sources, e.g. luminal, hormonal and neuronal. GLUTag cells are directly glucose-sensitive in the sub-millimolar concentration range, with half-maximum stimulation evident at ~0.2 mm glucose [21]. The detection mechanism involves both the closure of adenosine triphosphate (ATP)-sensitive potassium channels and the uptake of glucose by Na^+^-coupled glucose transporters. However, as GLUTag cells are currently the only *in vitro* L-cell model in which glucose dependence has been clearly demonstrated, the importance of direct glucose sensing by L-cells *in vivo*, and the relative roles of K_ATP_ channels vs. Na^+^-coupled glucose transporters or other glucose-sensing mechanisms, remain to be established.

The currents generated by Na^+^-coupled glucose transporters are particularly small relative to those that flow through ion channels, because in a transporter the ionic movement is directly coupled to the flux of substrate (e.g. two Na^+^ ions per glucose molecule transported). Other related transporters similarly couple the influx of a variety of nutrients, including amino acids and di-/tri-peptides, to Na^+^ or proton gradients. These uptake mechanisms play important roles in the absorptive properties of the gut, because they enable nutrients to be actively pumped out of the lumen against their concentration gradients. The idea that they might also be involved in cell signalling is more unusual. However, in GLUTag cells, Na^+^-coupled glutamine and asparagine transport can also be shown to trigger small depolarizing currents associated with GLP-1 secretion [22], and the idea that Na^+^-dependent glucose uptake can directly trigger GLP-1 release is supported by previous observations that non-metabolizable substrates of the intestinal glucose transporters trigger GLP-1 release from perfused intestinal preparations. It is therefore possible that L-cells *in situ* could sense luminal nutrients via the small depolarizing currents generated by nutrient influx at their apical membranes, generating electrical signals that could be directly coupled to hormone release.

## Physiological relevance

The incretin effect, which is responsible for up to 50% of the normal release of insulin following oral glucose ingestion, is significantly reduced in Type 2 diabetes [23], due both to the diabetes itself and to concomitant obesity [24]. This is believed to be a consequence of the impaired GLP-1 secretion described above, combined with reduced responsiveness of the islets to GIP. Interestingly, the insensitivity to GIP appears to be a consequence of receptor down-regulation at the β-cell plasma membrane, and recent reports have suggested that it may be reversible upon restoration of normoglycaemia [25]. Stimulating the release of incretin hormones might therefore be a rational potential therapeutic strategy in Type 2 diabetes, provided that the entero-endocrine axis is not severely impaired by ongoing poor glycaemic control. Indeed, the success of certain forms of bariatric surgery in lowering glucose levels in diabetic subjects, as well as in reducing appetite more generally, is widely attributed to the enhanced physiological release of entero-endocrine hormones such as GLP-1 and PYY [26]. Taken together with the known clinical effectiveness of GLP-1 mimetics and DPP-4 inhibitors in Type 2 diabetes, the data suggest that exploring the opportunities to stimulate the entero-endocrine axis *in vivo* might reveal exciting new therapeutic possibilities. Identifying and targeting G-protein-coupled receptors expressed specifically in the intestinal L-cells will be exciting areas for future drug development.

## Abbreviations

ATP: adenosine triphosphate
cAMP: cyclic adenosine monophosphate
DPP-4: dipeptidyl peptidase 4
GIP: glucose-dependent insulinotropic polypeptide
GLP-1: glucagon-like peptide-1
